# Sudden Sensorineural Hearing Loss as a Rare Sequela After Complete Recovery From COVID-19 Infection: Case Series and Literature Review

**DOI:** 10.7759/cureus.31856

**Published:** 2022-11-24

**Authors:** Rahaf Altwairqi, Mohammad H Shaheen, Albaraa Y Alsini, Bassam R Al-zuraiqi, Khalid Badr, Saeed Abdullah M Alghamdi, Elsaeid Mohamed Thabet Ali-Eldin, Fares Alghamdi

**Affiliations:** 1 Otolaryngology - Head and Neck Surgery, Al Hada Military Hospital, Taif, SAU; 2 Otolaryngology - Head and Neck Surgery, Al-Noor Specialist Hospital, Makkah, SAU; 3 Otolaryngology - Head and Neck Surgery, King Abdullah Medical City, Makkah, SAU; 4 Otolaryngology, King Abdullah Medical City, Makkah, SAU

**Keywords:** viral infection, sensorinerual hearing loss, intra-tympanic steroid, sudden sensorineural hearing loss (ssnhl), covid-19

## Abstract

Respiratory disease caused by a mutant coronavirus variant has spread rapidly worldwide. According to reports, the COVID-19 version propagated at the end of 2019 and originated in Wuhan, China. On January 30, 2022, the World Health Organization declared the outbreak a Public Health Emergency of International Concern, and on March 11, 2020, the outbreak has declared a pandemic. The COVID-19 infection might appear with no symptoms, very few symptoms, or extremely severe symptoms

We are the first to identify sudden sensorineural hearing loss (SSNHL) as a side effect in COVID-19 patients who have fully recovered from the illness. Additionally, all reported cases of this presentation have an unexplained unilateral left ear involvement. This article reviews the literature and four cases of COVID-19 patients with SSNHL.

We present four cases of COVID-19 positivity that were verified by PCR analysis of nasopharyngeal swabs. After fully recovering from the infection, all patients developed acute sensorineural hearing loss on the left side.

A deterioration in the hearing ability among COVID-19 survivors makes it possible that the problem persists long after their recovery from infection. To support such a claim, additional in-depth research is required. The current study, in our opinion, will contribute to an increase in understanding about COVID-19, promote awareness, and alert healthcare professionals to take into account and discuss any symptoms.

## Introduction

Respiratory disease caused by a mutant coronavirus variant has spread rapidly worldwide. According to reports, the COVID-19 version propagated at the end of 2019 and originated in Wuhan, China. On January 30, 2022, the World Health Organization declared the outbreak a Public Health Emergency of International Concern, and on March 11, 2020, the outbreak has declared a pandemic [1]. The COVID-19 infection might appear with no symptoms, very few symptoms, or extremely severe symptoms [2,3].

According to COVID-19's latent period, the symptoms may manifest 2-14 days after exposure [4]. Patients with COVID-19 commonly experience fever, coughing, exhaustion, gastrointestinal distress, and a loss of smell sensibility [5]. The aged and individuals with comorbidities are prone to disease and have unfavorable results [6]. Patients with COVID-19 disease experience various outcomes; some fully recover from infection without needing therapy, while others develop acute respiratory distress that necessitates admission to the intensive care unit [7].

Viral infections can result in unilateral or bilateral hearing loss, ranging in severity from minor to profound [8]. The literature describes some pathophysiology for hearing loss, including secondary to host inflammatory response and direct viral inner ear injury [9]. Typically, the hearing loss brought on by viral infection is sensorineural, while various types of hearing loss have been linked to certain viruses [10]. However, nasopharyngeal or throat swabs show that some COVID-19 patients have positive viral RNA even though they are asymptomatic [11]. It is still unknown if COVID-19 can affect the auditory system, even though many viruses have the potential to do so [11].

Sudden hearing impairment can be sensorineural or conducive. The latter condition is the sudden sensorineural hearing loss (SSNHL). SSNHL patients typically have no known cause; however, malignancies of the cerebellopontine angle, vascular diseases, autoimmune disorders, or viral infections have been implicated in some instances. More than 30 dB SNHL on more than three frequencies simultaneously for longer than three days meet the requirements to be considered SSNHL [12]. However, clinical work and academic research take hearing loss levels lower than 30 dB into account [12]. SSNHL management recommendations aim to find and address any underlying causes. Steroid therapy may aid in regaining hearing capacity [12]. If the systemic method is not employed or for salvage therapy, steroids can be administered intra-tympanic or systemically.

We are the first to identify SSNHL as a side effect in COVID-19 patients who have fully recovered from the illness. Additionally, all reported cases of this presentation have an unexplained unilateral left ear involvement. This article reviews the literature and four cases of COVID-19 patients with SSNHL.

## Case presentation

We present four cases of COVID-19 positivity that were verified by PCR analysis of nasopharyngeal swabs. After fully recovering from the infection, all victims developed acute sensorineural hearing loss on the left side (Table 1).

**Table 1:** Summary of the cases

Patient serial number	Age in years	Sex	Other otological symptoms	Level of sensorineural hearing loss
1	43	Male	Yes	Mild to moderate
2	40	Male	Yes	Mild to moderate
3	44	Female	No	Mild to moderate
4	35	Male	No	Mild to moderate

Case 1

A 43-year-old male patient is not known to have any chronic illness. The patient had to get a nasopharyngeal swab since they had been close to the COVID-19-positive patient. PCR analysis of the swab confirmed a positive result. He experienced chest pain, a cough, and a high-grade temperature while isolated at home. After total healing and the cessation of the symptoms mentioned above, he experienced intermittent tinnitus in his left ear for a month. When he abruptly lost hearing on his left side for a day, the patient went to the doctor. There was no history of recent upper respiratory tract infections, fever, ear pain, or discharge. The patient denied having ever had a chronic ear condition, had ear surgery, or used ototoxic drugs. Systemic oral steroids (Prednisolone) were administered to the patient for one week at a dosage equal to 1 mg/kg of body weight before being gradually weaned off over the following week. Tinnitus and hearing loss remained unchanged.

The patient then brought his problems to the otology clinic. The examination of the throat, nose, and ears revealed nothing exceptional. The Weber test was lateralized to the right ear using a tuning fork with a frequency of 512 Hz, and the Rinne test was positive in both ears. In both ears, tympanometry was type A. The left ear's sensorineural hearing loss was discovered by pure tone audiometry to be moderate at low frequencies and mild at high frequencies. On the other ear, the hearing thresholds were within normal ranges. After receiving two intratympanic injections of dexamethasone at a dose of 10 mg/mL separated by three days, the situation improved. The tinnitus vanished, and the patient's left ear's hearing fully returned to normal thresholds.

Case 2

A 40-year-old male patient is not known to have any chronic illness. He experienced lethargy and fever, and a nasopharyngeal swab revealed a COVID-19 infection. The patient was told to stay in seclusion at home till his condition got better. He experienced rapid left-side hearing loss with tinnitus and aural fullness two months after fully recovering from this infection. No prior history of fever, ear pain, or an upper respiratory illness during the past few weeks. The man denied having ever had chronic ear conditions, had ear surgery, or taken ototoxic drugs. The patient had systemic oral steroid therapy (Prednisolone) for five days at a dose of 60 mg daily, followed by a taper of 10 mg daily for an additional five days, but his hearing sense did not improve. He arrived at the otology clinic complaining of these symptoms. Examining the ear, nose, and throat revealed no abnormal findings. The results of the Weber test were lateralized to the right ear, while the Rinne test was positive in both ears. Tympanometry indicated bilateral type A. Pure tone audiometry indicated normal hearing thresholds on the right side and mild-moderate sensorineural hearing impairment on the left. A 4 mg/mL intratympanic dexamethasone injection was available as a form of treatment (three injections with three-day intervals between them). The patient's left ear recovered hearing to normal thresholds, while tinnitus and auditory fullness improved.

Case 3

A forty-four-year-old female patient has no history of any chronic disease. She is a doctor specializing in obstetrics and gynecology and has close relationships with some COVID-19-positive victims. The patient underwent a nasopharyngeal swab for COVID-19 after complaining of headaches, changes in taste and smell, and a loss of hearing. After a positive sample, the patient was told to stay home in isolation until complete recovery. She experienced sudden hearing loss on her left side for two months. There was no history of fever, tinnitus, auditory fullness, ear discharge, or ear pain. The patient had no history of ototoxic drug use, chronic ear conditions, or ear surgery. She received treatment with a systemic oral steroid (Prednisolone), 60 mg orally daily for five days, followed by a 10-mg daily decrease over the following five days.

After that, she reported to the otology clinic that her left ear's hearing had diminished. Complete ear, nose, and throat exams revealed nothing unusual. The Weber test was lateralized to the right ear, and Rinne's test was positive in both ears. Tympanometry was used to evaluate the hearing, and it found that both ears were type A. Except for a dip to 30 dB on 4k Hz, pure tone audiometry revealed normal hearing thresholds on the right side and mild to moderate sensorineural hearing impairment on the left side. One intratympanic dexamethasone injection (10 mg/mL) was given to the patient. She reported significant improvement, and her left ear's hearing thresholds had returned to normal levels before the infection.

Case 4

A 35-year-old male patient is known to have hypertension, polycythemia vera, and obstructive sleep apnea. While returning to Saudi Arabia during the COVID-19 epidemic, the patient had a nasopharyngeal swab performed. Despite the patient's denial of any symptoms at the time of the swab, the swab was detected as positive for COVID-19 by PCR. Without experiencing any COVID-19-related symptoms, the patient finished a two-week isolation period. He experienced a sudden hearing loss in his left ear for two days after two weeks of isolation. The patient rejected the presence of any additional otologic symptoms, a history of chronic ear conditions, past ear surgery, or usage of ototoxic drugs.

During the evaluation, the Rinne test was positive in both ears, and a tuning fork set to 512 Hz was used to lateralize the Weber test to the right ear. Tympanometry indicated type A in both ears. Pure tone audiometry showed normal hearing thresholds on the right side and mild-moderate sensorineural hearing loss on the left side. The patient was given the option of receiving an intra-tympanic dexamethasone injection due to his hypertension. At intervals of three days, patients received three 4 mg/mL injections. After the procedure, his left ear's hearing levels were restored to normal.

## Discussion

Encephalitis, meningitis, demyelination, and Guillain-Barre syndrome are a few of the neurologic symptoms linked to COVID-19 that have been reported [13]. Additionally, more cases of taste and smell abnormalities have been observed, raising the possibility that the COVID-19 virus has a direct neuropathic effect [13]. Potential neuropathic impacts on the vestibule-cochlear system may also occur [14]. A study tested the outer hair cells of asymptomatic COVID-19 victims. The control group showed increased high-frequency, pure-tone thresholds while transient evoked otoacoustic discharges were remarkably reduced in COVID-19 patients.

However, up to date, no specific treatment has been discovered yet, and understanding of this virus is also restricted. Being aware of the symptoms of COVID-19 will assist in establishing an early diagnosis and limit the disease's spread [15]. The ear, nose, and throat (ENT) symptoms were observed in some COVID-19 victims, especially younger women [16]. In general, the symptoms of COVID-19 victims may involve fever, cough, fatigue, gastrointestinal upset, and loss of smell [17]. The effect of COVID-19 is a captivating issue in audiology. An autopsic study of the middle ear and mastoid performed on three cadavers of COVID-19-positive patients showed that two out of three patients had positive COVID-19 on the middle ear swab. This finding warrants a potential COVID-19 invading the middle ear, and the possibility of inner ear invasion cannot be ruled out. A full precaution is essential in middle ear surgery as the virus may spread to the health care providers [18].

In the current investigation, we concentrate on SSNHL as a late sequela of the COVID-19 virus. According to the literature study, only a small number of people with COVID-19 illness developed SSNHL (Table 2). After fully recovering from the disease and its well-known repercussions, none of these patients experienced SSNHL. The first preliminary case of COVID-19 disease was reported in a senior female patient in Thailand, and there was no improvement in her hearing [19]. Sensorineural hearing loss in both ears in a patient hospitalized in the intensive care unit (ICU) with severe respiratory COVID-19 infection [20]. The patient was given two ototoxic drugs in the ICU. However, not all the patients in the current series were given them. A case of SSNHL was diagnosed in a COVID-19 patient in Turkey by Kilic et al. [21]. The patient had a complete hearing resolution on day 11 without steroid administration, though the hearing was assessed by phone as the patient was in isolation. Another case reported in Egypt was a male patient with a positive COVID-19 test. On day 3, the patient complained of left-side hearing loss. The audiometric test revealed significant SSNHL. The patient did not completely recover his hearing despite receiving the intratympanic steroidal injection. An adult patient with COVID-19 was recently hospitalized in the ICU and required 30-day intubation in a case reported in the UK [22]. The patient reported left-side hearing loss and tinnitus a week after being extubated. Despite receiving intratympanic steroid injections and oral steroids, the patient's PTA indicated left-side SHL [23].

**Table 2:** Reported cases of hearing loss related to COVID-19 infection

Author	# of cases	Comorbidity	Presentation	Degree of hearing loss	Affected ear	Other otological symptoms	Treatment by steroid	Complete hearing recovery
Sriwijitalai, et al. [19]	1	Not mentioned	Before resolution	Not mentioned	Not mentioned	Not mentioned	Not mentioned	No
Kilic, et al. [21]	1	Not mentioned	Before resolution	Not mentioned	Not mentioned	Not mentioned	No	Not mentioned
Rhman, et al. [22]	1	Not mentioned	Before resolution	Severe SNHL	Left	Yes	Yes	No
Degen, et al. [20]	1	No	Before resolution	Right: deafness Left: SNHL	Bilateral	Yes	Yes	No
Koumpa, et al. [23]	1	Asthma	Not mentioned	Severe SNHL	Left	Not mentioned	Yes	N
Present study	4	Only the patient in case number 4 has hypertension, polycythemia vera, and obstructive sleep apnea	After resolution	Mild- Moderate SNHL	Left	Yes	Yes	Yes

All four cases in this case series are left-sided SSNHLs that developed after full recovery from COVID-19 infection. The severity of the hearing loss ranged from mild to severe, and there was no clear gender predominance. Two out of four cases had other otological symptoms. There is no explanation in the literature for why the left ear was afflicted in every case reported in this series. The treatment with intratympanic dexamethasone injections produced outstanding results in all four described cases. Dexamethasone was used in 4 or 10 mcg/mL concentrations, depending on what was available. Less frequent injections of higher concentrations of dexamethasone were needed to treat the patients. There were no side effects from dexamethasone injections administered intratympanically.

## Conclusions

A deterioration in the hearing ability among COVID-19 survivors makes it possible that the problem persists long after their recovery from infection. To support such a claim, additional in-depth research is required. The current study, in our opinion, will contribute to an increase in understanding about COVID-19, promote awareness, and alert healthcare professionals to take into account and discuss any symptoms.
